# Quantitative CMR perfusion imaging identifies reduced flow reserve in microvascular coronary artery disease

**DOI:** 10.1186/1532-429X-18-S1-P79

**Published:** 2016-01-27

**Authors:** Peter W Shaw, Yang Yang, Kelvin Chow, Jorge A Gonzalez, Pelbreton C Balfour, Craig H Meyer, Fred Epstein, Jamieson Bourque, Michael Salerno, Christopher M Kramer

**Affiliations:** 1 Cardiology, University of Virginia, Charlottesville, VA USA; 2 Biomedical Engineering, University of Virginia, Charlottesville, VA USA; 3 Radiology, University of Virginia, Charlottesville, VA USA

## Background

Patients with angina without obstructive coronary artery disease are increasingly recognized and microvascular disease (MVD) is thought to play a significant role. An abnormal myocardial perfusion reserve (MPR) by positron emission tomography is predictive of increased cardiovascular events, particularly in women, diabetics, and those with metabolic syndrome. We hypothesized that MPR as measured by CMR would be reduced in patients with angina or anginal equivalent symptoms and non-obstructive CAD/normal coronaries compared with asymptomatic controls.

## Methods

Patients (women, diabetics or those with the metabolic syndrome) with suspected microvascular disease and no significant epicardial stenosis (≥50%) by x-ray angiography underwent vasodilator stress CMR perfusion studies on a 1.5T Siemens Avanto. Subjects without cardiovascular risk factors were recruited as controls and underwent the same vasodilator stress CMR study. High resolution first-pass spiral perfusion pulse sequences were performed. Sequence parameters included: 8 interleaves of variable density spirals from 0.75 to 0.2 Nyquist, 6.1 ms readout per interleaf, TE 1.0 ms, TR 9 ms, TI 80 ms, FA 35, FOV 340 mm, in-plane resolution 1.48 mm. Perfusion images were acquired during rest and stress at 3 short axis slice locations. Images were reconstructed by SPIRIT and then aligned with non-rigid registration ANTs (Advanced Normalization Tools). Quantification of perfusion was performed on a pixel-wise basis using Fermi-function deconvolution.

## Results

24 patients with MVD (20 female, 4 male, age (mean ± SD) 56 ± 13) completed the stress CMR protocol and were compared to 9 control subjects (6 F, 3 M, age 47 ± 13). Patients with MVD were predominantly Caucasian (79%) with an average BMI of 32.3 ± 6.6 and had hypertension (75%), dyslipidemia (88%), current or prior tobacco use (67%) and diabetes (25%). Rest flow was 1.28 ± 0.31 mL/min/g and stress flow 2.69 ± 0.64 mL/min/g with a resultant MPR of 2.15 ± 0.44. In controls, rest flow was 1.23 ± 0.25 mL/min/g and stress flow 3.35 ± 0.61 mL/min/g with MPR of 2.75 ± 0.32 (p < 0.001 vs. patients). Both subendocardial and subepicardial flow reserve were lower in patients than controls (p < 0.001) (Table 1). Rest flow (p < 0.01) and MPR (p < 0.05) were lower in diabetics. In hypertensive patients, rest and stress flows were lower (p < 0.05) but MPR was similar.

**Table 1:** Flow Values

	Rest Flow (mL/min/g)	Stress Flow (mL/min/g)	MPR
MVD (n = 24)	1.28 ± 0.31	2.69 ± 0.64	2.15 ± 0.44*
Controls (n = 9)	1.23 ± 0.25	3.35 ± 0.61	2.75 ± 0.32

*p < 0.001 vs. controls

## Conclusions

Patients with MVD and no significant epicardial coronary disease have reduced global MPR compared to asymptomatic controls as demonstrated by quantitative CMR perfusion imaging, likely due, in part, to endothelial dysfunction. This may contribute to their chest pain syndrome and adverse cardiovascular prognosis. Quantitative CMR perfusion imaging is a promising approach for its identification.

